# Analysis of Cembrane-Type Diterpenoids from Cultured Soft Coral *Sclerophytum flexibile* for Inhibition of TGF-β-Induced IL-6 Secretion in Inflammation-Associated Cancer

**DOI:** 10.3390/ijms262311280

**Published:** 2025-11-21

**Authors:** Yi-Chen Wang, Shun-Ban Tai, Jenq-Lin Yang, Pei-Feng Liu, Ping-Jyun Sung, Jui-Hsin Su, Chun-Lin Chen

**Affiliations:** 1 Division of Cardiology, Department of Internal Medicine, Kaohsiung Armed Forces General Hospital, Kaohsiung 802301, Taiwan; cvyc.wang@gmail.com; 2 Institute of Medical Science and Technology, National Sun Yat-sen University, Kaohsiung 80424, Taiwan; 3 Division of Cardiology, Department of Internal Medicine, Tri-Service General Hospital, National Defense Medical University, Taipei 100050, Taiwan; 4 Division of Rheumatology, Immunology and Allergy, Department of Internal Medicine, Zuoying Armed Forces General Hospital, Kaohsiung 81342, Taiwan; taishunban@gmail.com; 5 Department of Marine Biotechnology and Resources, National Sun Yat-sen University, Kaohsiung 80424, Taiwan; 6 Institute for Translational Research in Biomedicine, Kaohsiung Chang Gung Memorial Hospital, Kaohsiung 83301, Taiwan; jyang@cgmh.org.tw; 7 Department of Biomedical Science and Environmental Biology, Kaohsiung Medical University, Kaohsiung 80756, Taiwan; pfliu@kmu.edu.tw; 8 National Museum of Marine Biology and Aquarium, Pingtung 94450, Taiwan; pjsung@nmmba.gov.tw; 9 Graduate Institute of Marine Biology, National Dong Hwa University, Pingtung 974301, Taiwan; 10 Department of Biological Sciences, National Sun Yat-sen University, Kaohsiung 80424, Taiwan; 11 Department of Biotechnology, Kaohsiung Medical University, Kaohsiung 80708, Taiwan; 12 Graduate Institute of Natural Products, College of Pharmacy, Kaohsiung Medical University, Kaohsiung 80756, Taiwan

**Keywords:** cembranoids, TGF-β, intracellular trafficking

## Abstract

Cembrane-type diterpenoids (cembranoids), natural compounds derived from soft coral *Sclerophytum flexibile*, exhibit diverse biological activities including anti-inflammatory, anti-cancer, and anti-viral effects. Our previous research demonstrated that Sinulariolide, a member of this group, effectively inhibits TGF-β-induced IL-6 secretion, thereby suppressing inflammation-associated cancer development. Building on these findings, the present study employs a structure-activity relationship (SAR) approach to compare the anti-inflammatory properties of various cembranoids extracted from cultured soft coral *Sclerophytum flexibile*—a sustainable and environmentally friendly source that offers a consistent supply for research and therapeutic development. By isolating multiple cembrane-type analogs and analyzing their structural differences, we identified key chemical features that enhance their ability to interfere with TGF-β signaling and subsequent IL-6 production. The SAR analysis revealed distinct variations in anti-inflammatory efficacy among the tested compounds, pinpointing structural motifs crucial for inhibiting TGF-β-induced IL-6 secretion. These insights deepen our understanding of the molecular basis behind the anti-inflammatory action of cembranoids and guide the optimization of these compounds for potential therapeutic use.

## 1. Introduction

Emerging evidence highlights the pivotal role of both acute and chronic inflammatory processes in cancer development [1]. Inflammation has been implicated in various stages of cancer progression, including the initiation of neoplastic transformation, tumor growth, metastasis, and recurrence [2]. Advanced stages of cancer are often characterized by elevated levels of cytokines such as TGF-β, known for its immunosuppressive properties, and IL-6, which has immunostimulatory effects. These cytokines are associated with poor patient prognosis and are central to creating a tumor microenvironment conducive to epithelial-to-mesenchymal transition (EMT) and metastatic spread [3,4,5]. Additionally, they enhance tumor cell resistance to chemotherapy [6].

Although traditionally regarded as an anti-inflammatory molecule, TGF-β actively shapes the inflammatory milieu within tumors by promoting tissue remodeling and inhibiting antigen-specific CD8^+^ T cell activity in the local tumor microenvironment [7]. Research has shown that TGF-β induces IL-6 gene expression across various cell types, including prostate cancer cells, fibroblasts, osteoblasts, and retinal pigmented epithelial cells [8,9,10,11,12,13,14,15]. Elevated IL-6 levels, in turn, contribute significantly to the oncogenic potential of TGF-β, as demonstrated in prostate tumorigenesis [8]. These findings underscore the interplay between TGF-β and IL-6 in driving tumor progression while counteracting the growth-suppressive effects of TGF-β.

Current adjuvant therapies seek to counteract the pro-tumorigenic signals of TGF-β by curbing its ligand production, neutralizing its activity, or inhibiting its receptor kinase function. However, because TGF-β plays essential roles in maintaining normal tissue function, direct inhibition often leads to serious side effects such as cardiovascular issues and benign tumor growth [16]. These findings suggest the potential of targeting non-canonical (non-Smad) TGF-β pathways as a more effective and safer strategy, particularly when combined with approaches that modulate IL-6 production. By strategically intervening in these interconnected signaling networks, future therapies may achieve improved efficacy and safety in the treatment of cancer.

**Figure 1:**
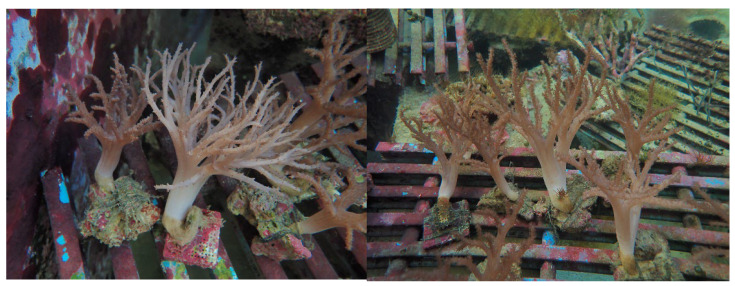
The cultured soft coral *Sclerophytum flexibile*.

Cultured soft coral-derived natural products exhibit a diverse range of bioactive properties, including anti-inflammatory, antibacterial, antiviral, and anti-cancer activities [17]. Among these, cembrane-type diterpenoids (cembranoids)—abundantly produced by the soft coral *Sclerophytum flexibile* (Figure 1)—have attracted significant attention due to their potent biological effects. Our previous studies have shown that sinulariolide (**1**), an active compound extracted from cultured *Sclerophytum flexibile*, inhibits TGF-β-induced interleukin-6 production by modulating the p38 MAPK-NF-κB pathway in lung and liver cancer models [18]. Additionally, sinulariolide suppresses the growth of melanoma, hepatoma, and lung adenocarcinoma cell lines through mechanisms involving mitochondrial pathways and endoplasmic reticulum stress [19,20,21,22]. Other cembranoids, such as 11-dehydrosinulariolide (**2**) [23], 3,4:8,11-bisepoxy-7-acetoxcembra-15(17)-en-1,12-olide (**5**) [24], and sinularin (**9**) [25], exhibit weak to moderate cytotoxicity against various cancer cell lines. These compounds also demonstrate significant anti-inflammatory effects by inhibiting the accumulation of pro-inflammatory cytokines (interleukin (IL)-6, IL-12, and tumor necrosis factor-α), inducible nitric oxide synthase (iNOS) and COX-2 proteins in lipopolysaccharide-stimulated RAW264.7 macrophage cells [26].

This study identifies key structural features of cembranoids from *Sclerophytum flexibile* that inhibit TGF-β-induced IL-6 secretion, revealing variations in anti-inflammatory efficacy. The findings enhance understanding of their molecular mechanisms and support their optimization for therapeutic use, with *S. flexibile* providing a sustainable source for future drug development.

## 2. Results

### 2.1. Cytotoxicity of Cembranoids

**Figure 2:**
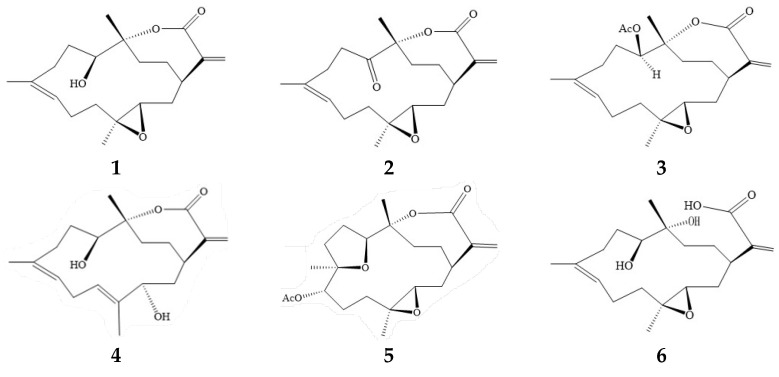

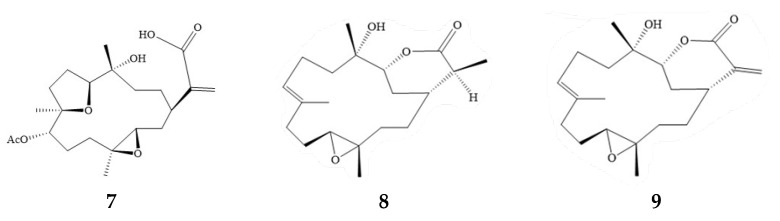
Structures of cembrane-type diterpenoids **1**–**9**.

The chemical structures and related information for cembranoids, sinulariolide (**1**), 11-dehydrosinulariolide (**2**), 11-epi-sinulariolide acetate (**3**), (−)-sandensolide (**4**) 3,4:8,11-bisepoxy-7-acetoxcembra-15(17)-en- 1,12-olide (**5**), flexibilin A (**6**), dendronpholide F (**7**), dihydrosinularin (**8**) and sinularin (**9**), are presented in Figure 2. To rule out the possibility that reduced cytokine production was due to cembranoid-induced cell death, A549 and HepG2 cells were tested for viability at various cembranoid concentrations. The CCK-8 assay revealed that exposing cells to up to 40 μM of cembranoids for 48 h did not result in significant cell death (IC_50_ > 40 μM). Because the concentrations used in this study did not exceed 20 μM, the observed changes in protein expression and luciferase activity following cembranoid and TGF-β treatment cannot be attributed to alterations in cell number or viability. Moreover, morphological assessments of A549 cells treated with 40 μM cembranoids for 24 h showed no detectable morphological changes in response to cembranoids. Based on compound availability during purification and their low cytotoxicity, **1** and **5**–**9** were selected for further activity and mechanistic studies.

### 2.2. Cembranoids Differentially Inhibit TGF-β-Induced IL-6 Expression in Cultured Cells

**Figure 3:**
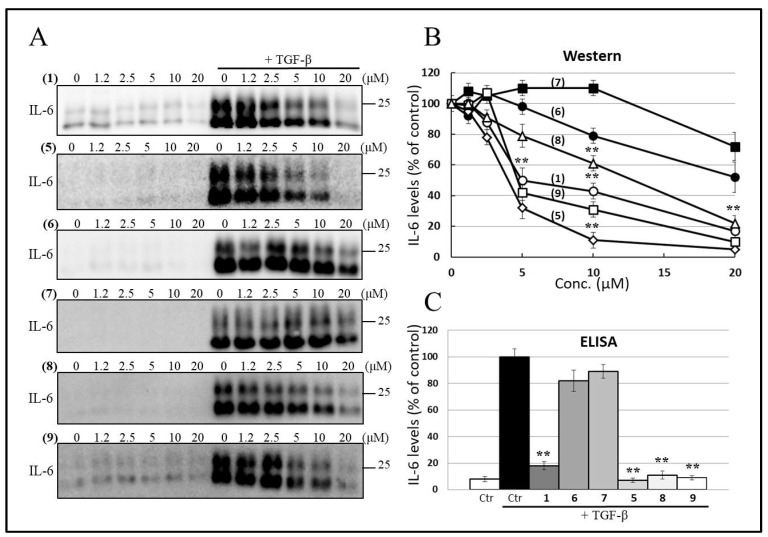
Cembranolide analogs inhibit TGF-β-induced IL-6 synthesis in A549 cells. (**A**) Western blots of conditioned media show reduced IL-6 after treatment with sinulariolide (**1**), 3,4:8,11-bisepoxy-7-acetoxycembra-15(17)-en-1,12-olide (**5**), flexibilin A (**6**), dendronpholide F (**7**), dihydrosinularin (**8**), and sinularin (**9**); equal loading was verified by Coomassie Brilliant Blue (CBB) staining of matched aliquots (see Supplementary Figure S1). (**B**) Densitometric quantification from three independent blots (mean ± SD, Supplementary Figure S1A–F); individual blots and analyses are shown in the Supplementary Materials (IL-6 CM Westerns for compounds #1, #5, #6, #7, #8, #9; repeats 1–3). (**C**) ELISA using distinct anti-IL-6 antibodies confirms inhibition of TGF-β-induced IL-6 secretion. For all panels, cells were serum-starved, pretreated with 0–20 μM cembranoids for 4 h, then stimulated with 200 pM TGF-β for 48 h; final DMSO was ≤0.01% (*v*/*v*) and matched across conditions. Data are mean ± SD (*n* = 3); ** *p* < 0.001.

We first evaluated and compared the inhibitory effects of cembranoids on TGF-β-induced IL-6 production in A549 cells. A549 and HepG2 cells were selected for this study due to their high endogenous TβRII expression and the absence of mutations or loss in other key components of the TGF-β signaling pathway [27,28]. Our previous findings indicated that (**1**) (Figure 2) inhibits TGF-β-induced IL-6 production without affecting EMT or causing morphological changes. Western blot analysis confirmed that TGF-β stimulation significantly increased IL-6 secretion into the culture medium (Figure 3A, lane 7 vs. lane 1). To assess whether cembranoids could suppress TGF-β-induced IL-6 secretion in comparison to (**1**), A549 cells were pretreated with (**1**), (**5**), (**6**), (**7**), (**8**), and (**9**) before TGF-β stimulation. Among these, (**1**), (**5**), (**8**), and (**9**) significantly inhibited IL-6 upregulation in a dose-dependent manner (Figure 3A, lanes 7–12). Quantitative analysis (Figure 3B) revealed the following order of inhibitory potency based on IC_50_ values: (**5**) (IC_50_: 3.5 μM) > (**9**) (IC_50_: 4.2 μM) > (**5**) (IC_50_: 4.8 μM) > (**8**) (IC_50_: 10.0 μM) > (**6**) (IC_50_ > 20.0 μM) > (**7**) (IC_50_ > 20.0 μM), “TGF-β alone with vehicle” set to 100% (=3, Supplementary Figure S1A–F: IL-6 CM Westerns for compounds #1, #5, #6, #7, #8, #9; repeats 1–3). Additionally, IL-6 protein levels in A549 culture supernatants were measured using ELISA following TGF-β and cembranoid treatments. As shown in Figure 3C, untreated A549 cells secreted 210 pg/mL of IL-6, which increased to 2354 pg/mL upon 200 pM TGF-β stimulation (set as 100%, *p* < 0.001). Treatment with (**1**), (**5**), (**8**), and (**9**) (20 μM for 48 h) led to a significant decrease in IL-6 levels, achieving inhibition rates of 81%, 91%, 88%, and 89%, respectively (**, *p* < 0.001). (**6**) and (**7**) exhibited moderate inhibition of 19% and 10%, respectively.

### 2.3. Cembranoid Inhibition of TGF-β-Induced IL-6 mRNA Expression

**Figure 4:**
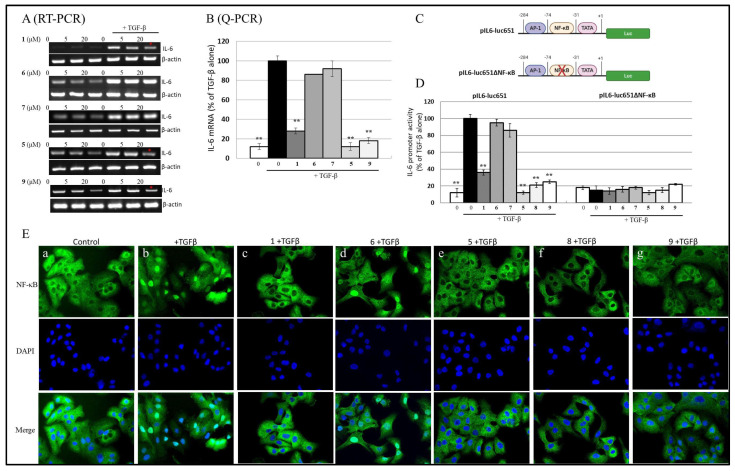
Cembranolides Inhibit TGF-β-Stimulated NF-κB Nuclear Translocation and IL-6 Production. (**A**) Gel-resolved RT-PCR and (**B**) qRT-PCR analysis demonstrating the inhibitory effects of cembranolides on TGF-β-induced IL-6 mRNA expression. A549 cells were pretreated with cembranolides before being stimulated with TGF-β for 24 h. IL-6 mRNA levels were assessed via RT-PCR, with red asterisks marking compounds that significantly reduced IL-6 expression. (** *p* < 0.001). (**C**) Schematic representation of IL-6 luciferase promoter constructs, including a wild-type construct and a truncated version lacking the NF-κB binding site. (**D**) Effects of cembranolides on TGF-β-induced IL-6 transcriptional activity. The wild-type IL-6 promoter was significantly activated by TGF-β, whereas the NF-κB-truncated promoter construct showed no response to TGF-β stimulation. (** *p* < 0.001). Data are representative of three independent experiments. (**E**) Immunofluorescence analysis of NF-κB nuclear translocation in A549 cells treated with cembranolides (**1**, **6**, **5**, **8**, and **9**). In (**a**–**g**), Cells were pretreated with 20 μM cembranolides for 4 h, followed by 30 min of TGF-β stimulation. NF-κB localization was detected using monoclonal antibodies, highlighting the impact of cembranolides on TGF-β-induced NF-κB activation.

Our previous work demonstrated that (**1**) reduced TGF-β-stimulated IL-6 mRNA production in A549 and HepG2 cells. In this study, we compared the effects of other cembranoids on TGF-β-induced IL-6 mRNA expression using gel-resolving RT-PCR (Figure 4A) and qRT-PCR (Figure 4B) under similar experimental conditions. Figure 4A shows IL-6 mRNA from cells treated with 100 pM TGF-β for 8 h, with the expected sizes being 565 bp for IL-6 cDNA and 225 bp for β-actin, which served as an internal control. The RT-PCR and qRT-PCR results indicated that TGF-β up-regulated IL-6 mRNA and that (**1**), (**5**), and (**9**) specifically inhibited IL-6 mRNA production (red asterisks indicates decreased significantly), while (**6**) and (**7**) had no effect. Consistently, the qRT-PCR data in Figure 4B confirmed that TGF-β increased IL-6 mRNA levels, which were significantly down-regulated by (**1**), (**5**), and (**9**) treatment (** *p* < 0.001).

### 2.4. Cembranoids Suppress TGF-β-Induced IL-6 Expression via NF-κB-Dependent Promoter Activity

**Figure 5:**
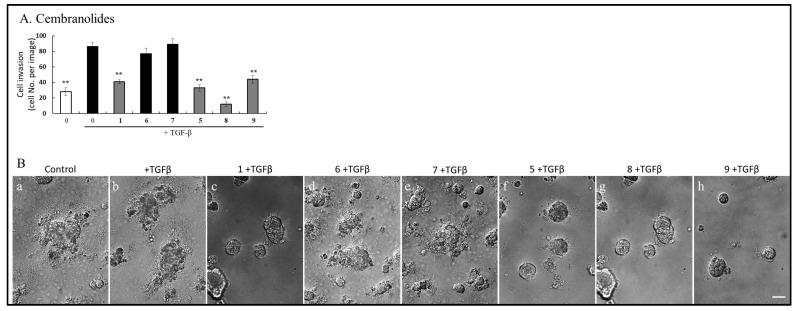
Cembranolides Inhibit TGF-β-Stimulated Migration and Invasion in A549 Cells. A549 cell migration and invasion were assessed using Transwell and spheroid invasion assays. (**A**) In the Transwell Migration Assay, A549 cells suspended in serum-free medium containing cembranolides were seeded into the upper chambers of Matrigel-coated filter inserts and allowed to adhere for 1 h, followed by treatment with or without 200 pM TGF-β for 24 h. Migrated cells were stained, and the number of invaded cells per image was quantified. Data are presented as mean ± SD from three independent experiments (** *p* < 0.001 vs. TGF-β-treated group). (**B**) In the Spheroid Invasion Assay, representative images of A549 spheroids on day 14 of the extracellular matrix (ECM) invasion assay are shown, with the spreading edges indicated as invasion (scale bar: 1 mm). In (**a**–**h**), A549 spheroids were embedded in Matrigel plugs within serum-free medium containing with or without cembranolides and incubated with or without 200 pM TGF-β for 14 days to evaluate the inhibitory effects of cembranolides on TGF-β-induced invasion.

The impact of cembranoids on TGF-β-stimulated IL-6 production was evaluated using luciferase reporter assays. A 651-bp fragment of the human IL-6 promoter was cloned into a luciferase vector, generating the pIL6-luc651 construct (Figure 4C). When transfected into A549 cells, treatment with 100 pM TGF-β increased pIL6-luc651 activity to 240 ± 12% relative to the unstimulated control (set as 100%). The addition of 20 μM (**1**), (**5**), (**8**), and (**9**) reduced TGF-β-mediated IL-6 promoter activation to 62%, 89%, 79%, and 77%, respectively (** *p* < 0.001) (Figure 4D). In contrast, (**6**) and (**7**) did not affect IL-6 promoter activity, which is consistent with the mRNA and protein expression data. Notably, in cells transfected with a reporter construct lacking the NF-κB binding site (pIL6-luc651∆NF-κB) [29], TGF-β treatment did not alter IL-6 promoter activity. NF-κB is a key regulatory factor in IL-6 gene transcription, and its activation by various cytokines is mediated through the p38 MAPK pathway in many human cell types [18,30,31]. As shown in Figure 4C, transient transfection of the mutant IL-6-luc construct, which lacks the NF-κB binding element, resulted in a significant reduction in TGF-β-induced luciferase activity compared to the wild-type IL-6-luc construct. To assess the inhibitory effects of different cembranoids on TGF-β-mediated NF-κB activation, we examined their ability to suppress TGF-β-induced nuclear translocation of NF-κB. Immunofluorescence microscopic analysis in A549 cells revealed that TGF-β stimulation led to a marked nuclear translocation of NF-κB (Figure 4E). However, pretreatment with (**1**), (**5**), (**8**), and (**9**) completely inhibited this nuclear translocation (Figure 5B(c) vs. Figure 5B(b)), while (**6**) and (**7**) [18] exhibit no inhibitory effect. These findings suggest that TGF-β activates NF-κB via the p38 MAPK pathway in lung cancer cells and that (**1**), (**5**), (**8**), and (**9**) effectively suppress TGF-β-induced IL-6 secretion through inhibition of the p38-NF-κB signaling cascade.

### 2.5. Cembranoids Blocked TGF-β-Induced Cancer Cell Invasion

IL-6 has been implicated in promoting multiple aspects of tumor progression, including cell proliferation, epithelial-mesenchymal transition (EMT), migration, invasion, and metastasis [32]. Based on this, we investigated whether cembranoid-mediated inhibition of IL-6 expression could suppress TGF-β-induced cancer cell invasion. The effects of cembranoids on cell invasion were evaluated using transwell invasion and cancer spheroid invasion assays. For the transwell invasion assay, cells were pretreated with 20 μM of **1, 5**, **7**, **8**, and **9** for 4 h, followed by 24 h stimulation with TGF-β. Compared to the DMSO-treated control group, TGF-β significantly enhanced cell invasion (Figure 5A, quantitative results). (**1**), (**5**), (**8**), and (**9**) treatment effectively suppressed TGF-β-induced invasion at a concentration of 20 μM (Figure 5A). Similarly, in the cancer spheroid invasion assay performed in Matrigel, (**1**), (**5**), (**8**), and (**9**) significantly reduced the number of A549 cells invading the surrounding matrix compared to TGF-β-treated cells (Figure 5B(c,f–h) vs. Figure 5B(b)). In contrast, (**6**) and (**7**) did not exhibit inhibitory effects (Figure 5B(d,e)). These findings suggest that cembranoids suppress the migratory and invasive capacity of cancer cells in vitro, potentially through IL-6 inhibition.

## 3. Discussion

**Figure 6:**
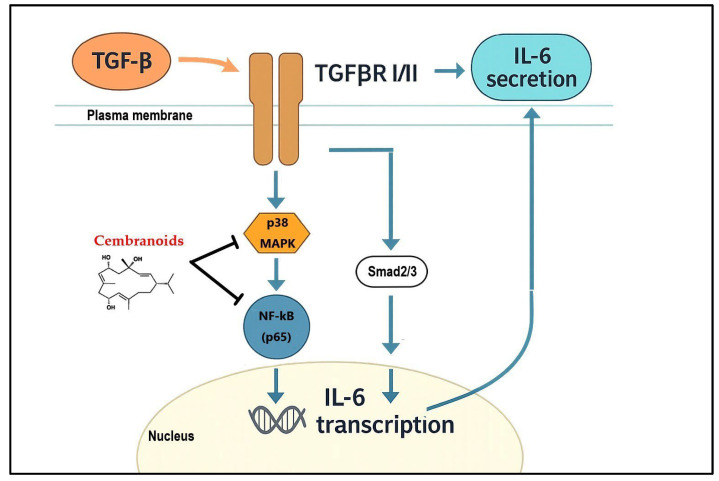
Mechanistic schematic of cembranoid action on TGF-β driven inflammation. TGF-β engagement activates TGF-β RI/II and bifurcates into Smad2/3 (maintained) and non-Smad p38 → IKK → NF-κB pathways. Cembranoids (**1**, **5**, **8**, **9**) preferentially dampen the p38 → NF-κB arm, lowering IL-6 promoter activity, IL-6 mRNA, and secreted IL-6 while sparing the canonical Smad branch.

The structural diversity of cembranoids—especially 1 and 5–9—governs their activity against TGF-β-induced IL-6. Compounds **1**, **5**, **8**, and **9** are most potent, whereas 6 and 7 are weaker, consistent with greater steric bulk reducing target engagement. Activity tracks with lactone architecture (six- or seven-membered; notably strong in 8/9), favorable acetylation (e.g., 5 ≫ 7, implicating improved binding or stability), and macrocyclic conjugation, while molecular flexibility/sterics further tune efficacy (Table 1). These features collectively enhance inhibition of IL-6—likely via the p38 MAPK → NF-κB arm—thereby dampening tumor-promoting inflammation and migration (Figure 6). This SAR framework (lactone ring, acetyl modification, lower steric hindrance, macrocycle effects) provides a clear basis for structure-guided optimization of cembranoids targeting TGF-β–driven pathology. Importantly, all compounds tested did not affect canonical Smad signaling under TGF-β stimulation (e.g., p-Smad2/3, SBE-luc), consistent with our prior study [18]; to avoid redundancy, we note this selectivity in the Methods/Discussion rather than repeating negative controls throughout the figures. Prior work on cembranoids such as sinularin and flexibilide has largely reported broad anti-inflammatory/anticancer actions—e.g., NF-κB or ERK/JNK modulation, growth inhibition, apoptosis, and anti-migration—using composite endpoints (COX-2/iNOS, ROS, viability, generic NF-κB readouts) rather than a single, disease-relevant cytokine loop. In contrast, our series of nine congeners defines actionable SAR, showing that a six-membered lactone framework with favorable acetylation and lower steric bulk (**1**, **5**, **8**, **9** ≫ **6**, **7**) tracks with potency against TGF-β–driven IL-6. Notably, effective concentrations (10–20 μM) suppress IL-6 with low-to-moderate cytotoxicity, indicating selective disruption of a tumor-promoting cytokine circuit rather than nonspecific growth inhibition.

Cembranoids suppress TGF-β-induced IL-6 while sparing canonical Smad signaling, indicating selective inhibition of non-Smad, tumor-promoting inflammation [8,13,14,15]. This selectivity is valuable given TGF-β’s dual role in cancer, where broad blockade can be deleterious. Prior studies show a reciprocal TGF-β↔IL-6 loop across multiple cell types (including PSCs and epithelial models), implicating IL-6 as a driver that amplifies TGF-β activity [15,33,34]. Our data therefore support cembranoids as intervention points to disrupt this loop, reducing IL-6 output without impairing essential Smad functions, and motivate further evaluation as anti-inflammatory, anti-cancer candidates.

Beyond cancer treatment, the therapeutic potential of cembranoids may extend to a broader range of inflammatory diseases. IL-6 is a key player in chronic inflammation and has been implicated in fibrosis, autoimmune disorders, and cardiovascular diseases. Given the ability of cembranoids to selectively modulate IL-6 secretion, future studies should investigate their efficacy in preclinical models of inflammatory diseases. This would provide further insight into their therapeutic potential and pave the way for their development as a novel class of anti-inflammatory agents with applications beyond oncology.

The development of new drugs often involves substantial costs associated with both chemical synthesis and the isolation of natural compounds from wild sources. These financial and environmental burdens highlight the need for more sustainable and economically viable drug discovery approaches. Cultured soft corals, such as *Sclerophytum flexibile*, offer a promising alternative by providing a controlled and renewable supply of valuable bioactive compounds. Through aquaculture techniques, these corals can be sustainably cultivated, reducing pressure on natural marine ecosystems while ensuring a consistent and scalable source of cembranoids for pharmaceutical research and development. Leveraging advances in coral aquaculture can facilitate the discovery of novel therapeutics while promoting environmental conservation and reducing production costs.

Cembranoids exhibit low to moderate cytotoxicity, making them particularly promising as complementary cancer treatments that focus on reducing inflammation rather than directly targeting tumor cells with cytotoxic mechanisms. Their anti-inflammatory effects are observed at physiologically relevant concentrations of 10–20 µM, effectively inhibiting IL-6 production without impairing cell viability. This concentration range offers a safer and more targeted strategy to modulate tumor-associated inflammation, potentially improving treatment efficacy while minimizing adverse effects when used alongside conventional cancer therapies.

Moving forward, several aspects warrant further investigation. First, in vivo validation in metastatic cancer models is essential to confirm the therapeutic potential of cembranoids [19,20,21,22,24,35,36,37,38,39,40]. Second, detailed mechanistic studies are needed to elucidate how cembranoids modulate other components of the tumor microenvironment, including immune cells and stromal fibroblasts. Third, combination studies with existing chemotherapy agents could provide insight into synergistic effects that enhance treatment efficacy while reducing toxicity.

In conclusion, cembranoids selectively suppress TGF-β-induced IL-6 while preserving canonical Smad signaling, coinciding with reduced cancer cell migration/invasion. This combination of non-Smad pathway modulation and low cytotoxicity supports their development as anti-inflammatory, anti-cancer leads. The next steps include structure-guided optimization, in vivo validation in metastatic models, and rational combinations with standard therapies to enhance efficacy.

## 4. Materials and Methods

### 4.1. Reagents and Antibodies

Cembranoids used in this study were isolated and characterized from the soft coral *Sclerophytum flexibile* by Prof. Jui-Hsin Su at the National Museum of Marine Biology and Aquarium in Pintung, Taiwan. These bioactive molecules include sinulariolide (**1**), 3,4:8,11-bisepoxy-7-acetoxcembra-15(17)-en-1,12-olide (**5**), flexibilin A (**6**), dendronpholide F (**7**), dihydrosinularin (**8**) and sinularin (**9**). Each compound was dissolved in DMSO to prepare 20 mM stock solutions, which were stored at −80 °C. In all experiments, the final DMSO concentration was maintained below 0.01%, ensuring that it did not interfere with TGF-β signaling [41]. Primary monoclonal antibodies against NF-κB p65 (#8242) and IL-6 (#12912) were obtained from Cell Signaling Technology (Beverly, MA, USA), while the β-actin antibody (ab8227) was sourced from Abcam Inc. (Cambridge, MA, USA). Secondary antibodies, including horseradish peroxidase-conjugated goat anti-mouse IgG and goat anti-rabbit IgG, were supplied by Millipore (catalog#MA5-23698, Billerica, MA, USA). 4,6-diamidino-2-phenylindole (DAPI) was purchased from Sigma Chemical Co. (St. Louis, MO, USA), and cell culture reagents were obtained from Invitrogen (Burlington, ON, Canada). Recombinant human TGF-β was acquired from R&D Systems (catalog#7754-BH, Minneapolis, MN, USA). The human IL-6 luciferase promoter constructs were generously provided by Dr. Chih-Hsin Tang (School of Medicine, China Medical University, Taichung, Taiwan). Additional chemicals and inhibitors were sourced from Sigma-Aldrich (St. Louis, MO, USA).

### 4.2. Extraction and Isolation of Cembrane Diterpenoids from S. flexibile Extract

Specimens of the soft coral *Sclerophytum flexibile* were initially collected off the coast of Pingtung, southern Taiwan, in June 2005. These specimens were transferred to an 80-ton cultivation tank equipped with a flow-through seawater system at the National Museum of Marine Biology and Aquarium. The material used in this research was harvested from the tank in October 2018, and a voucher specimen was subsequently deposited at the National Museum of Marine Biology and Aquarium, Taiwan. For chemical analysis, the collected coral was freeze-dried and minced, yielding a wet weight of 5.0 kg and a dry weight of 500 g. The dried material was exhaustively extracted at room temperature using ethyl acetate (EtOAc) in ten successive 4.0 L portions. The ethyl acetate extract was evaporated under reduced pressure to afford a residue (34.5 g), and the residue was subjected to column chromatography on silica gel, using *n*-hexane, *n*-hexane and EtOAc mixture of increasing polarity, and finally pure EtOAc to yield 10 fractions: Fr-1 (eluted by pure *n*-hexane), Fr-2 (eluted by *n*-hexane:EtOAc, 50:1), Fr-3 (eluted by *n*-hexane:EtOAc, 10:1), Fr-4 (eluted by *n*-hexane:EtOAc, 5:1), Fr-5 (eluted by *n*-hexane:EtOAc, 3:1), Fr-6 (eluted by *n*-hexane:EtOAc, 2:1), Fr-7 (eluted by *n*-hexane:EtOAc, 1:1), Fr-8 (eluted by *n*-hexane:EtOAc, 1:2), Fr-9 (eluted by *n*-hexane:EtOAc, 1:5), Fr-10 (eluted by pure EtOAc). The detailed protocol for purifying the compounds—fraction 5 was further purified with normal-phase HPLC (*n*-hexane:EtOAc, 2:1) to obtain **2** (5.5 mg) and **3** (10.5 mg). Fraction 6 was further separated by silica gel column chromatography with gradient elution (*n*-hexane:EtOAc, 2:1 to 1:1) to afford **1** (2 g) and **5** (1.5 g). Fraction 7 was separated by normal-phase HPLC using *n*-hexane:EtOAc (1:2) to afford **4** (8.5 mg), **8** (1.2 g) and **9** (1.2 g). Fraction 8 was separated by normal-phase HPLC using *n*-hexane:EtOAc (1:3) to afford **6** (106.2 mg) and **7** (97.5 mg).

### 4.3. Cell Culture

The A549 non-small-cell lung cancer cell line (CCL-185) and the HepG2 hepatocellular carcinoma cell line (HB-8065) were obtained from ATCC (Manassas, VA, USA) and cultured following the recommended protocols. Both cell lines were maintained in Dulbecco’s Modified Eagle’s Medium (DMEM) supplemented with 10% fetal bovine serum (FBS). Prior to experimentation, the cells were tested to confirm the absence of mycoplasma contamination and were used within 25 passages after thawing to ensure consistency and reliability of the results.

### 4.4. Conditioned Medium (CM) Preparation and Analysis

CM was collected from cells serum-starved for 48 h, clarified by centrifugation (300× *g*, 10 min, 4 °C), and stored at −80 °C. Proteins were concentrated by trichloroacetic acid (TCA) precipitation as described previously [42], pellets were resuspended in sample buffer, and total protein was quantified by BCA (Thermo Fisher Scientific, Waltham, MA, USA). After TCA precipitation, all CM samples were normalized to equal protein concentration and loaded at 20 µg per lane for Western blotting. Loading equivalence was confirmed by Coomassie Brilliant Blue staining of a duplicate gel run from the same normalized CM aliquots. IL-6 levels in CM were assessed by Western blot and by ELISA (Quantikine IL-6, R&D Systems, Carlsbad, CA, USA).

### 4.5. Western Blotting

For Western blot analysis, total protein was extracted from A549 and HepG2 cells using RIPA buffer supplemented with 25 μg/mL phenylmethylsulfonyl fluoride. The lysates were incubated on ice for 30 min and then centrifuged at 14,000 rpm for 10 min at 4 °C. Protein concentrations were determined using a BCA Protein Assay Kit. Equal amounts of protein (25 μg) were resolved on a 10% SDS-PAGE gel and subsequently transferred onto polyvinylidene difluoride (PVDF) membranes. Following a 2 h blocking step with 5% non-fat milk, membranes were incubated with primary antibodies at 4 °C for 24 h. Secondary antibodies conjugated to horseradish peroxidase were then applied at room temperature for 1 h. Immunoreactive bands were detected by enhanced chemiluminescence (ECL; Amersham Biosciences, Buckinghamshire, UK) and imaged on an ImageQuant™ LAS 4000 system (GE Healthcare, Chicago, IL, USA). Exposures were typically ~5–9 min at medium sensitivity, with settings adjusted to avoid saturation. Densitometry was performed in ImageJ (v1.43, NIH, Bethesda, MD, USA) using only unsaturated images within the linear range, and no selective manipulation was applied (only uniform, full-image adjustments as needed).

### 4.6. Immunofluorescent Staining

A549 cells were cultured on 25 mm coverslips and treated with 100 pM TGF-β, either alone or in combination with varying concentrations of cembranoids. Following treatment, the cells were fixed with 4% paraformaldehyde (PFA) for 15 min, then washed three times with cold PBS. Cells were permeabilized using 0.5% Triton X-100 for 10 min, followed by another three PBS washes. Blocking was performed with an immunofluorescence (IF) blocking buffer for 3 h before incubating with NF-κB antibody (1:800) in the same buffer for 2 h at room temperature. After primary antibody incubation, cells were exposed to a secondary antibody diluted in IF blocking buffer for 2 h at room temperature. The secondary antibody was subsequently removed, and cells were rinsed with PBS before being counterstained with 1 μg/mL DAPI for nuclear staining for 10 min at room temperature with gentle rocking. Coverslips were washed four times with PBS (10 min each) before imaging with the Thermo ArrayScan high-content imaging system. Nine fields per well were captured using two imaging channels: one for nuclear identification and quantification, and the other for measuring NF-κB expression. The fluorescence intensity ratio between the cytoplasm and nucleus was quantified to assess NF-κB translocation per cell. Data are expressed as mean values from three independent experiments.

### 4.7. Cell Viability

The cytotoxic effects of cembranoids were assessed using the Cell Counting Kit-8 (CCK-8; Dojindo, Tokyo, Japan) following the manufacturer’s instructions. A549 cells were seeded in 96-well plates and allowed to adhere for 24 h. Cells were then treated with cembranoids at concentrations of 2, 5, 10, 40, or 100 μM in FBS-free DMEM, alongside an untreated control group. After 48 h of incubation, 10 μL of CCK-8 working solution was added to each well, followed by a 1 h incubation at 37 °C. Optical density (OD) was measured at 450 nm using the SpectraMax ID3 (Molecular Devices, LLC, San Jose, CA, USA).

### 4.8. Enzyme-Linked Immunosorbent Assay (ELISA)

IL-6 protein levels in the culture supernatants were measured using an ELISA assay. A549 cells were seeded in 12-well plates and pre-treated with cembranoids for 4 h before being stimulated with TGF-β for 48 h. Following treatment, the supernatants were collected, and IL-6 protein concentrations were determined using the Quantikine IL-6 ELISA Kit (Boster Bio, Wuhan, China) according to the manufacturer’s instructions. Data were presented as mean ± SD, and statistical significance was evaluated using a *t*-test.

### 4.9. Transwell Invasion Assay

A549 and HepG2 cells were treated with 20 μM cembranoids, with or without TGF-β, for 24 h. A total of 2 × 10^4^ cells were suspended in serum-free medium and seeded into the upper chamber of a Transwell insert (Greiner, Kremsmünster, Austria) pre-coated with Matrigel. The lower chamber was filled with medium containing 10% FBS to serve as a chemoattractant, and the cells were incubated at 37 °C for 16 h. After incubation, the migrated cells were fixed with methanol and stained with crystal violet. Images were captured at ×200 magnification using a NIKON TE2000-U inverted microscope (Nikon, Tokyo, Japan), and the number of migrated cells was quantified using ImageJ software.

### 4.10. Cell Transfection and Luciferase Assays

A549 cells were cultured in 24-well plates (2 × 10^4^ cells/well), serum-starved for 24 h, and transfected using Lipofectamine 3000 (Thermo Fisher Scientific) for 2 h [29]. Following transfection, cells were overlaid with low-serum medium containing cembranoids at the specified concentrations or vehicle control. After a 4 h preincubation, cells were stimulated with 50 pM TGF-β for 24 h. Subsequently, cells were lysed, and equal amounts of lysates were subjected to firefly luciferase activity analysis using the Dual Luciferase Assay (Promega, Madison, WI, USA) according to the manufacturer’s protocol. Briefly, 100-μL aliquots of cell lysates were combined with 50 μL of luciferase reagent buffer, and luminescence was measured over a 10-s integration period using a SpectraMax ID3 (Molecular Devices, LLC, San Jose, CA, USA). To normalize transfection efficiency, Renilla luciferase expression driven by the thymidine kinase promoter (0.4 μg/well) was used as an internal control.

### 4.11. Reverse Transcription-Quantitative Polymerase Chain Reaction (RT-PCR) and Quantitative (qPCR)

RNA extraction was carried out using TRIzol reagent (Thermo Fisher Scientific). A total of 2 µg of RNA was reverse-transcribed into complementary DNA (cDNA) using M-MLV reverse transcriptase (Promega Corporation, Madison, WI, USA) in a 20 µL reaction mixture. The reaction contained M-MLV 5× Reaction Buffer, 10 mM dNTP, 500 µg/mL oligo dT15 primers, and nuclease-free water, followed by incubation at 42 °C for 15 min. Quantitative PCR (qPCR) was performed using 100 pg of cDNA as a template, with SYBR-Green PCR Master Mix (Thermo Fisher Scientific) in an ABI 7500 Fast Real-Time PCR system (Thermo Fisher Scientific). The primer sequences used were as follows: IL-6, forward 5′-ACATCGACCCGTCCACAGTAT-3′ and reverse 5′-CAGAGGGGTAGGCTTGTCTC-3′; β-actin, forward 5′-CGTGTTCCTACCCCCAATGT-3′ and reverse 5′-TGTCATCATACTTGGCAGGTTTCT-3′. β-actin served as the internal reference gene to normalize transcription levels, and relative fold changes were calculated in comparison to untreated controls.

### 4.12. Statistical Analysis

Statistical analysis was performed using one-way analysis of variance (ANOVA) to compare differences among multiple groups. Data are presented as the mean ± standard deviation (SD) from at least two to three independent experiments. A *p*-value of less than 0.001 was considered statistically significant.

## Figures and Tables

**Table 1:** Key SAR patterns affecting cembranoids activities. These structural features correlate with concordant decreases in IL-6 protein, mRNA, and promoter activity, and with blockade of NF-κB nuclear translocation, indicating inhibition of the p38–NF-κB arm while preserving Smad signaling.

Structural Feature	Representative Compounds	Activity vs. TGF-β-Induced IL-6	Notes/Mechanistic Readouts
Six-membered lactone framework within the macrocycle	**8**, **9**	Strong inhibition (ELISA ~88–89% at 20 µM)	Tracks with NF-κB reporter suppression and blocked NF-κB nuclear translocation; points to p38–NF-κB pathway engagement.
Acetylation (–AcO) on cembrane scaffold	**5** (vs. **7**)	5 ≫ 7 for IL-6 inhibition (5~91% at 20 µM; 7~10%)	Suggests acetylation can enhance receptor engagement/intracellular stability; placement/context matters (not merely presence).
Lower steric bulk/greater conformational fit	**1**, **5**, **8**, **9**	Consistently potent across protein, mRNA, and promoter levels	Bulky 6, 7 show weak or negligible effects (ELISA ~ 19%/10%; IC_50_ > 20 µM), consistent with reduced target engagement.
Macrocyclic conjugation & lactone presence (general)	**1**, **5**, **8**, **9**	Superior potency across assays	SAR indicates these motifs tune activity toward non-Smad IL-6 control via p38–NF-κB while sparing canonical Smad signaling.
Cytotoxicity window	All tested	Active at 10–20 µM with IC_50_ for viability >40 µM	Functional inhibition not attributable to cell loss; supports true pathway modulation.

## Data Availability

The original contributions presented in this study are included in this article/Supplementary Materials. Further inquiries can be directed to the corresponding authors.

## Supplementary Materials

The following supporting information can be downloaded at: https://www.mdpi.com/article/10.3390/ijms262311280/s1.
