# Supplementary material for: Analysis of Cembrane-Type Diterpenoids from Cultured Soft Coral Sclerophytum flexibile for Inhibition of TGF-β-Induced IL-6 Secretion in Inflammation-Associated Cancer
Source: Int J Mol Sci. 2025 Nov 21;26(23):11280. doi: 10.3390/ijms262311280 (PMC12692079; doi:10.3390/ijms262311280)
Supplement: Supplementary file 1 [file ijms-26-11280-s001.zip › ijms-3920857-supplementary.pptx]

## Slide 1
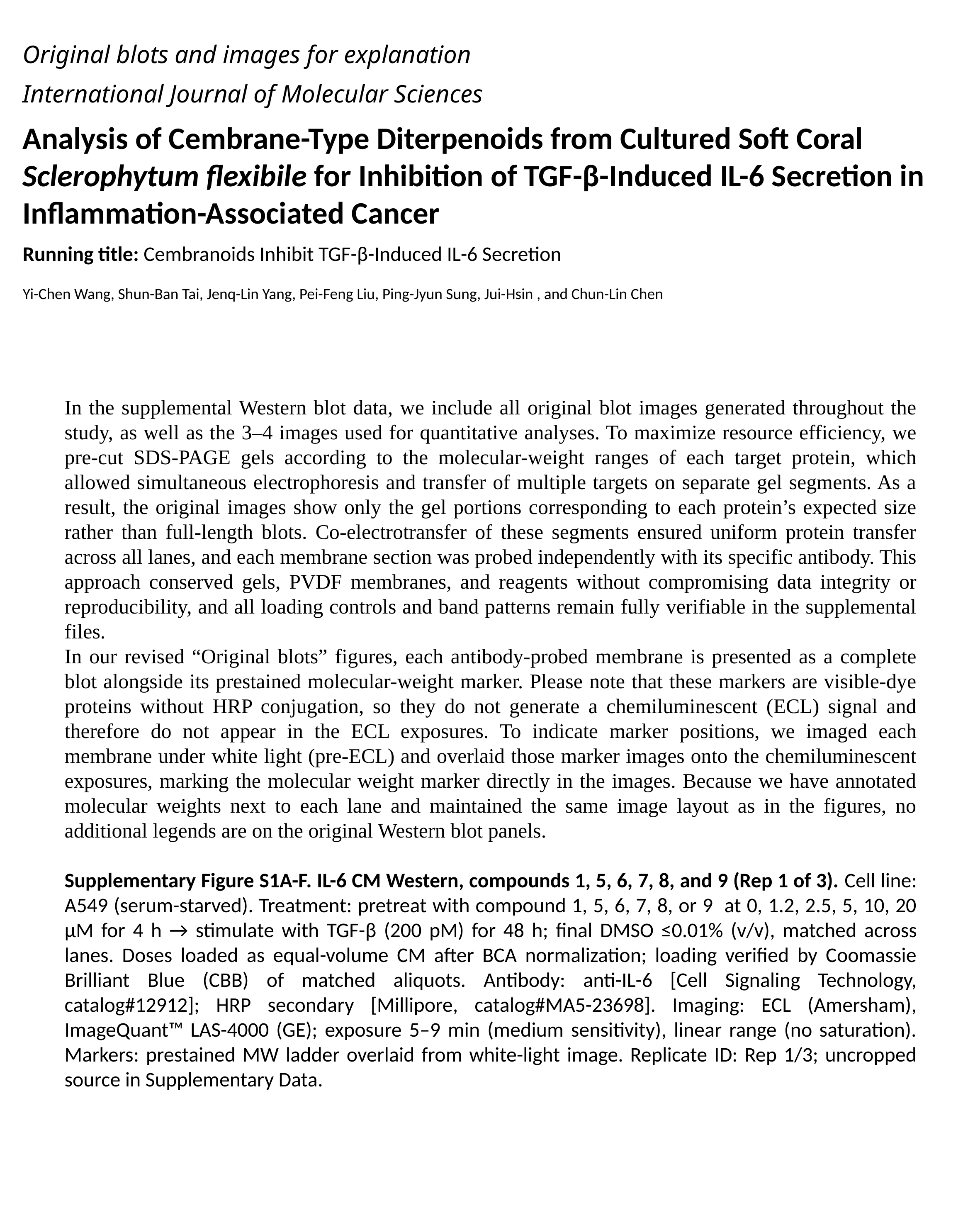

Original blots and images for explanation
International Journal of Molecular Sciences
Analysis of Cembrane-Type Diterpenoids from Cultured Soft Coral Sclerophytum flexibile for Inhibition of TGF-β-Induced IL-6 Secretion in Inflammation-Associated Cancer
Running title: Cembranoids Inhibit TGF-β-Induced IL-6 Secretion
Yi-Chen Wang, Shun-Ban Tai, Jenq-Lin Yang, Pei-Feng Liu, Ping-Jyun Sung, Jui-Hsin , and Chun-Lin Chen
In the supplemental Western blot data, we include all original blot images generated throughout the study, as well as the 3–4 images used for quantitative analyses. To maximize resource efficiency, we pre-cut SDS-PAGE gels according to the molecular-weight ranges of each target protein, which allowed simultaneous electrophoresis and transfer of multiple targets on separate gel segments. As a result, the original images show only the gel portions corresponding to each protein’s expected size rather than full-length blots. Co-electrotransfer of these segments ensured uniform protein transfer across all lanes, and each membrane section was probed independently with its specific antibody. This approach conserved gels, PVDF membranes, and reagents without compromising data integrity or reproducibility, and all loading controls and band patterns remain fully verifiable in the supplemental files.
In our revised “Original blots” figures, each antibody-probed membrane is presented as a complete blot alongside its prestained molecular-weight marker. Please note that these markers are visible-dye proteins without HRP conjugation, so they do not generate a chemiluminescent (ECL) signal and therefore do not appear in the ECL exposures. To indicate marker positions, we imaged each membrane under white light (pre-ECL) and overlaid those marker images onto the chemiluminescent exposures, marking the molecular weight marker directly in the images. Because we have annotated molecular weights next to each lane and maintained the same image layout as in the figures, no additional legends are on the original Western blot panels.
Supplementary Figure S1A-F. IL-6 CM Western, compounds 1, 5, 6, 7, 8, and 9 (Rep 1 of 3). Cell line: A549 (serum-starved). Treatment: pretreat with compound 1, 5, 6, 7, 8, or 9 at 0, 1.2, 2.5, 5, 10, 20 µM for 4 h → stimulate with TGF-β (200 pM) for 48 h; final DMSO ≤0.01% (v/v), matched across lanes. Doses loaded as equal-volume CM after BCA normalization; loading verified by Coomassie Brilliant Blue (CBB) of matched aliquots. Antibody: anti-IL-6 [Cell Signaling Technology, catalog#12912]; HRP secondary [Millipore, catalog#MA5-23698]. Imaging: ECL (Amersham), ImageQuant™ LAS-4000 (GE); exposure 5–9 min (medium sensitivity), linear range (no saturation). Markers: prestained MW ladder overlaid from white-light image. Replicate ID: Rep 1/3; uncropped source in Supplementary Data.

## Slide 2
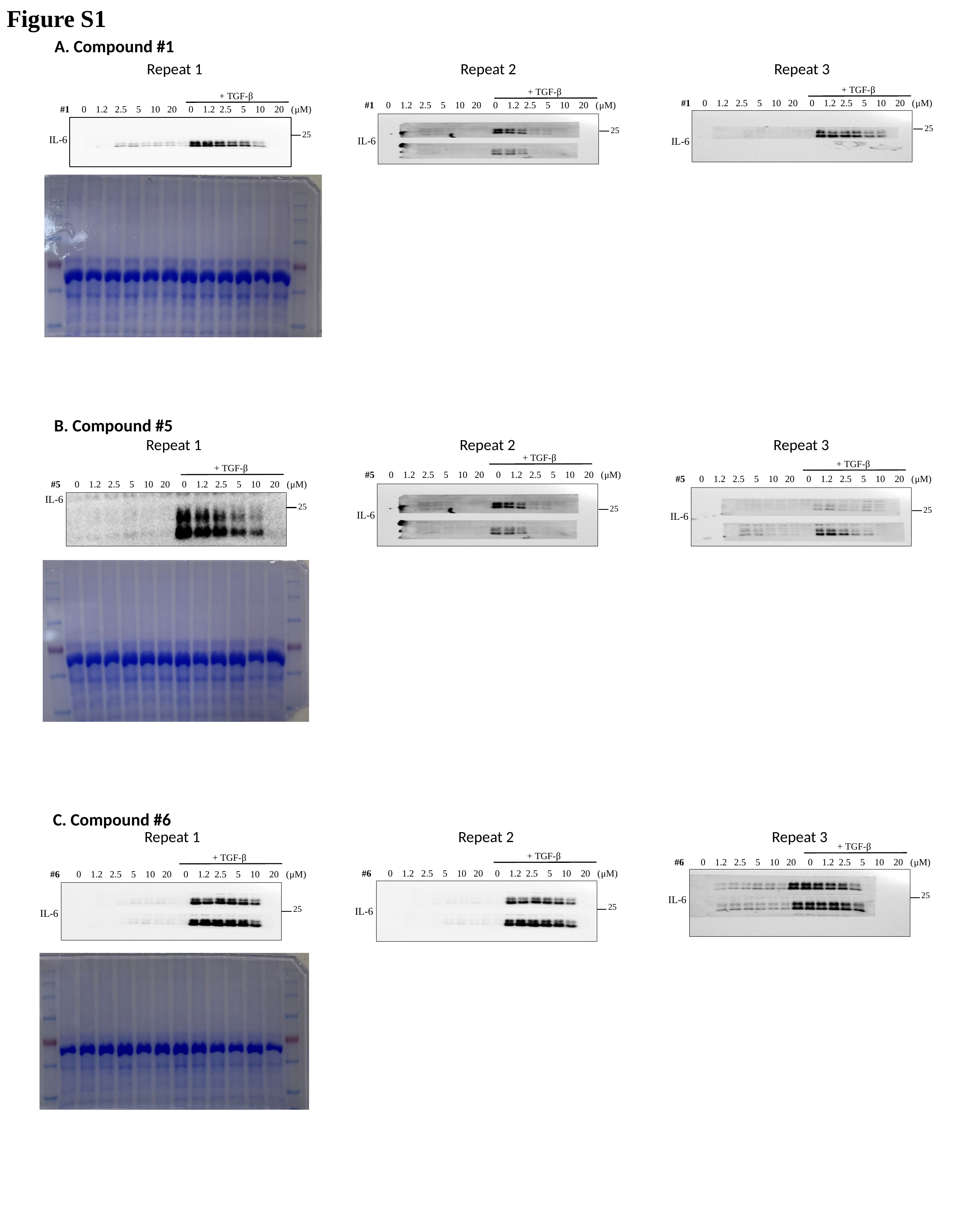

Figure S1
A. Compound #1
Repeat 1
Repeat 2
Repeat 3
+ TGF-β
+ TGF-β
+ TGF-β
#1 0 1.2 2.5 5 10 20 0 1.2 2.5 5 10 20 (μM)
#1 0 1.2 2.5 5 10 20 0 1.2 2.5 5 10 20 (μM)
#1 0 1.2 2.5 5 10 20 0 1.2 2.5 5 10 20 (μM)
25
25
25
IL-6
IL-6
IL-6
B. Compound #5
Repeat 1
Repeat 2
Repeat 3
+ TGF-β
+ TGF-β
+ TGF-β
#5 0 1.2 2.5 5 10 20 0 1.2 2.5 5 10 20 (μM)
#5 0 1.2 2.5 5 10 20 0 1.2 2.5 5 10 20 (μM)
#5 0 1.2 2.5 5 10 20 0 1.2 2.5 5 10 20 (μM)
IL-6
25
25
25
IL-6
IL-6
C. Compound #6
Repeat 1
Repeat 2
Repeat 3
+ TGF-β
+ TGF-β
+ TGF-β
#6 0 1.2 2.5 5 10 20 0 1.2 2.5 5 10 20 (μM)
#6 0 1.2 2.5 5 10 20 0 1.2 2.5 5 10 20 (μM)
#6 0 1.2 2.5 5 10 20 0 1.2 2.5 5 10 20 (μM)
25
IL-6
25
25
IL-6
IL-6

## Slide 3
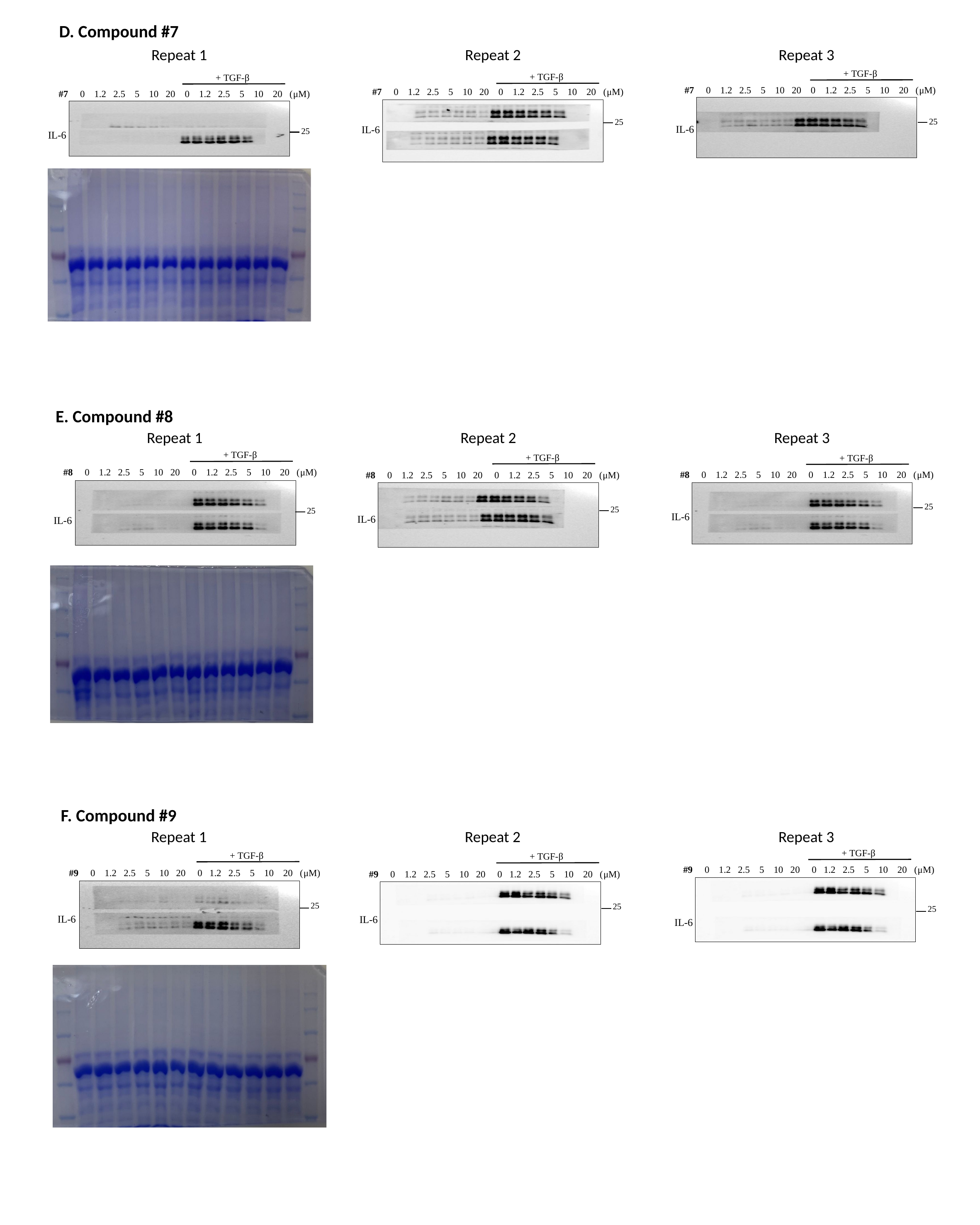

D. Compound #7
Repeat 1
Repeat 2
Repeat 3
+ TGF-β
+ TGF-β
+ TGF-β
#7 0 1.2 2.5 5 10 20 0 1.2 2.5 5 10 20 (μM)
#7 0 1.2 2.5 5 10 20 0 1.2 2.5 5 10 20 (μM)
#7 0 1.2 2.5 5 10 20 0 1.2 2.5 5 10 20 (μM)
25
25
IL-6
IL-6
25
IL-6
E. Compound #8
Repeat 1
Repeat 2
Repeat 3
+ TGF-β
+ TGF-β
+ TGF-β
#8 0 1.2 2.5 5 10 20 0 1.2 2.5 5 10 20 (μM)
#8 0 1.2 2.5 5 10 20 0 1.2 2.5 5 10 20 (μM)
#8 0 1.2 2.5 5 10 20 0 1.2 2.5 5 10 20 (μM)
25
25
25
IL-6
IL-6
IL-6
F. Compound #9
Repeat 1
Repeat 2
Repeat 3
+ TGF-β
+ TGF-β
+ TGF-β
#9 0 1.2 2.5 5 10 20 0 1.2 2.5 5 10 20 (μM)
#9 0 1.2 2.5 5 10 20 0 1.2 2.5 5 10 20 (μM)
#9 0 1.2 2.5 5 10 20 0 1.2 2.5 5 10 20 (μM)
25
25
25
IL-6
IL-6
IL-6
